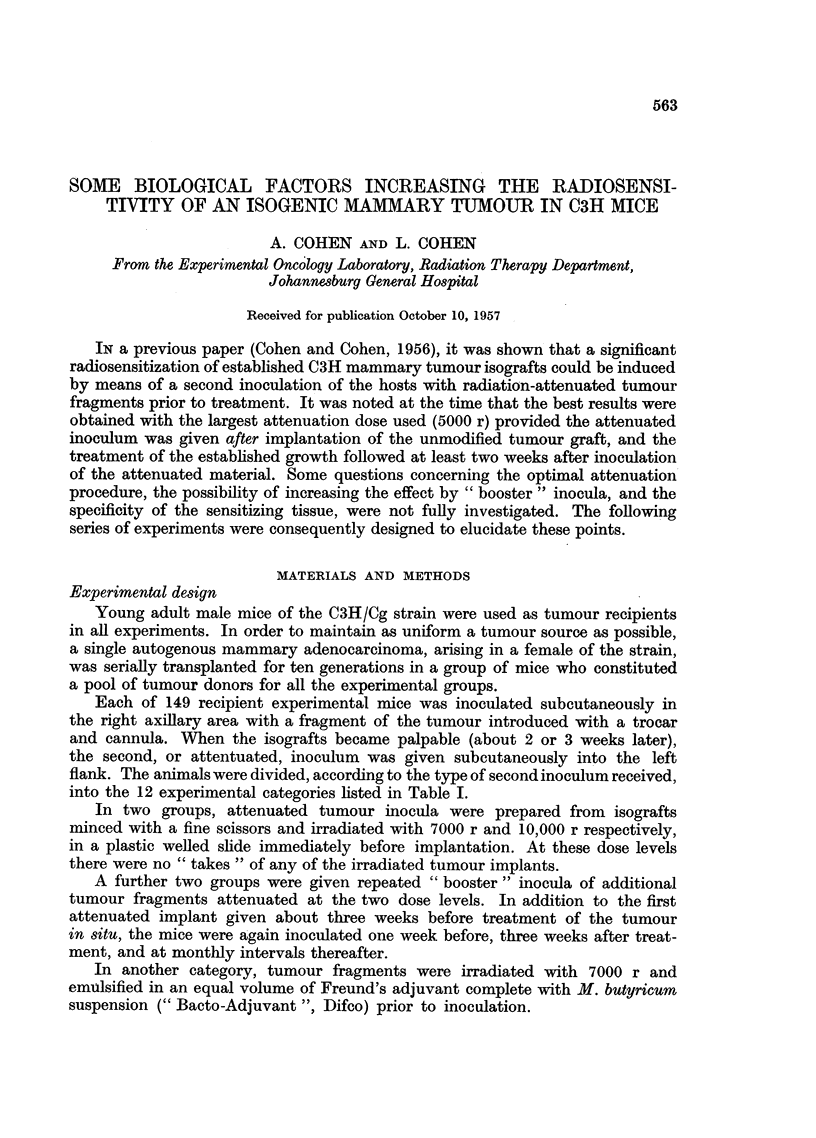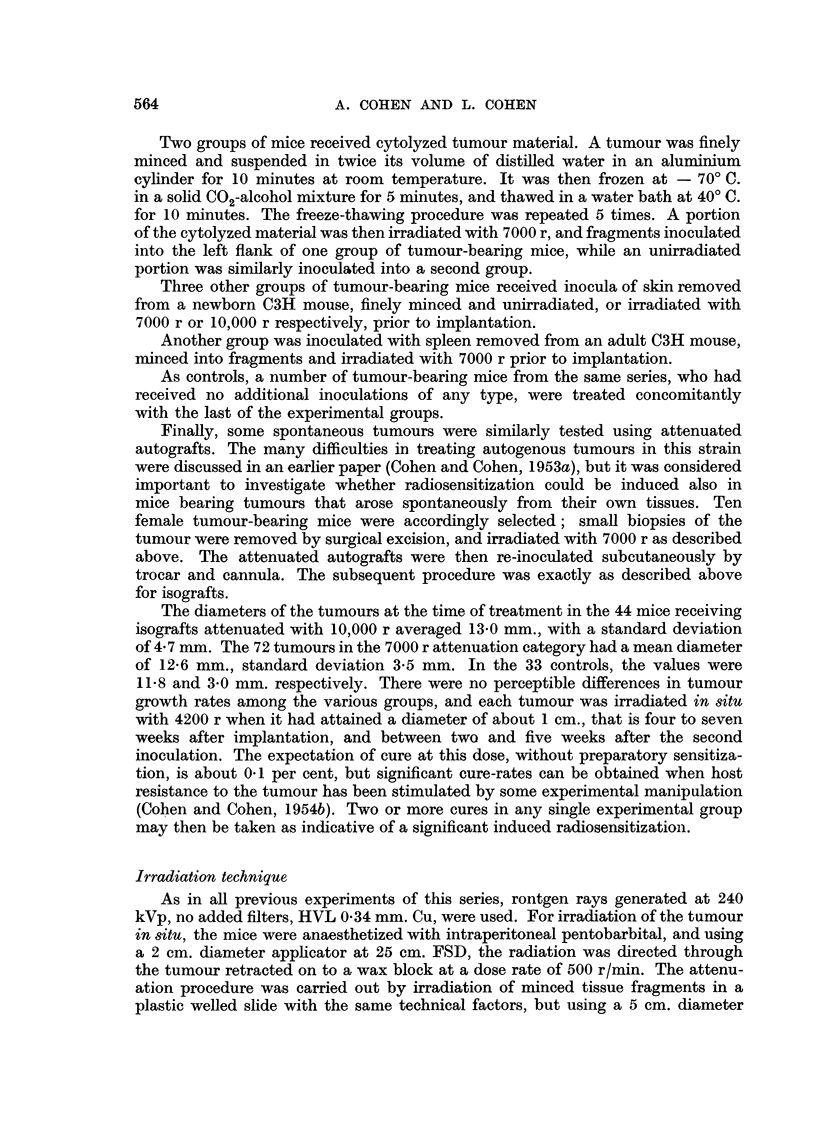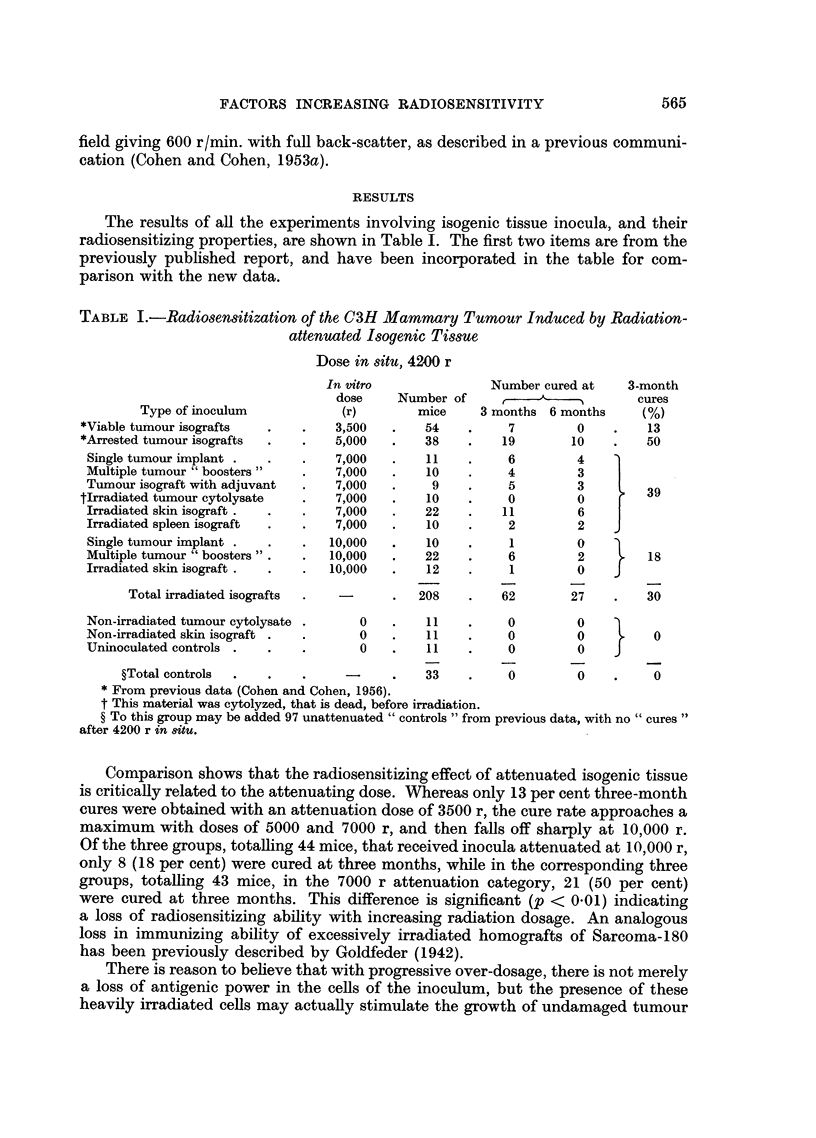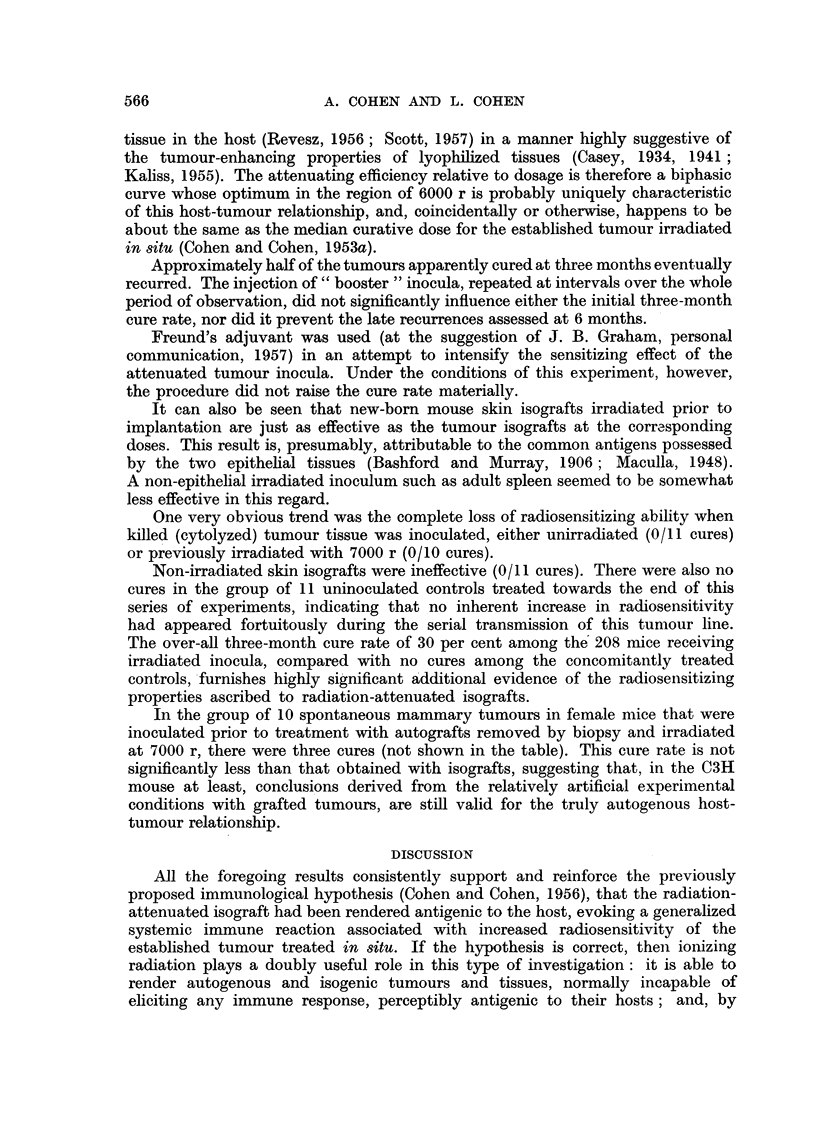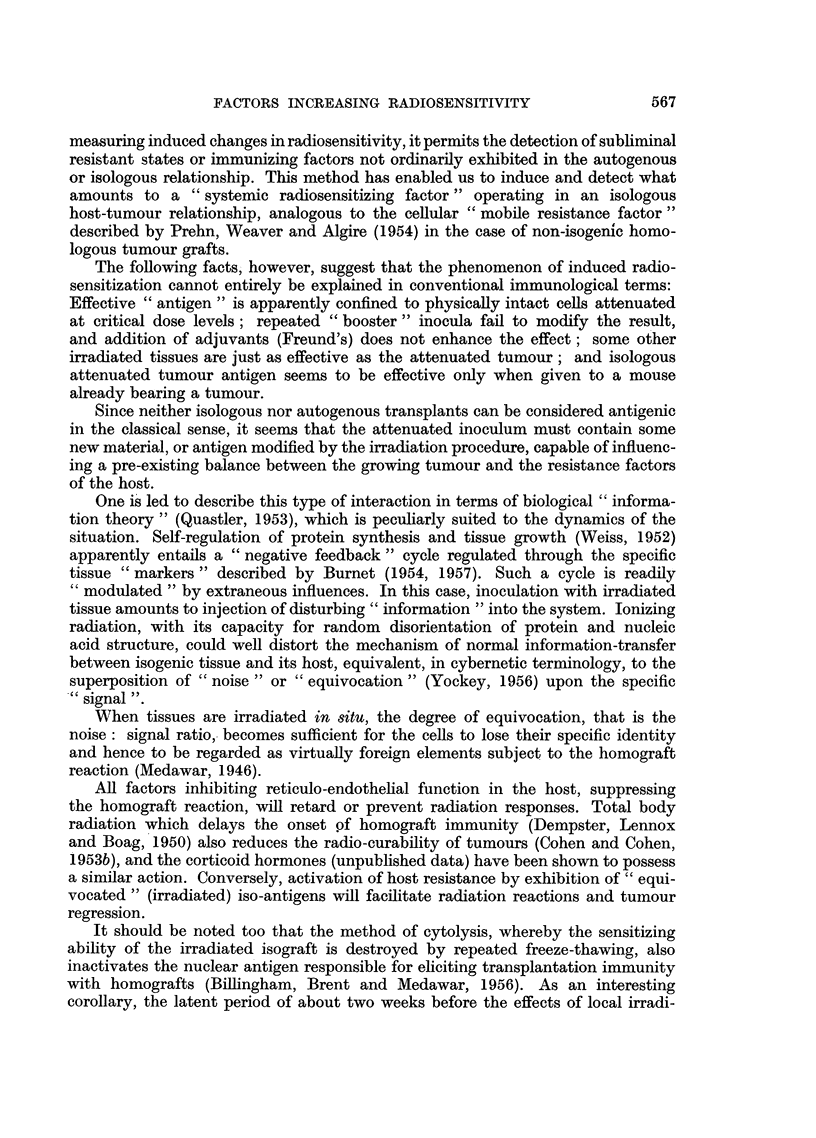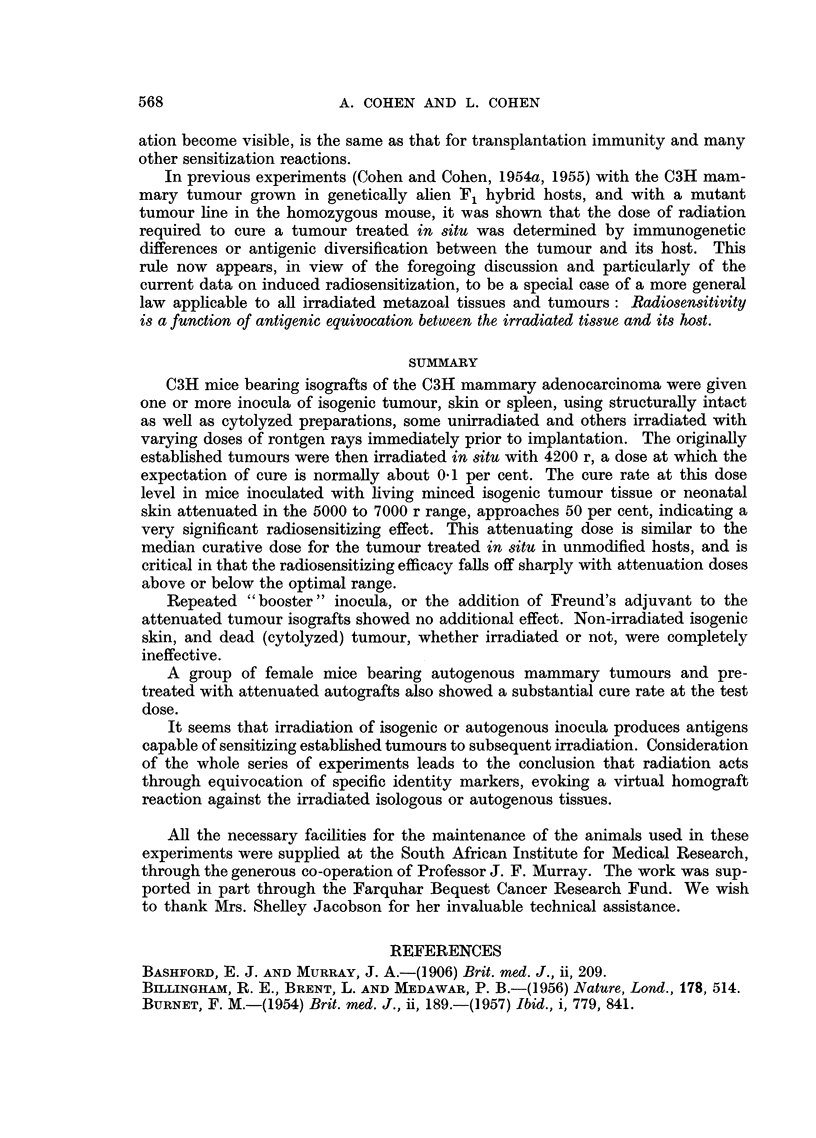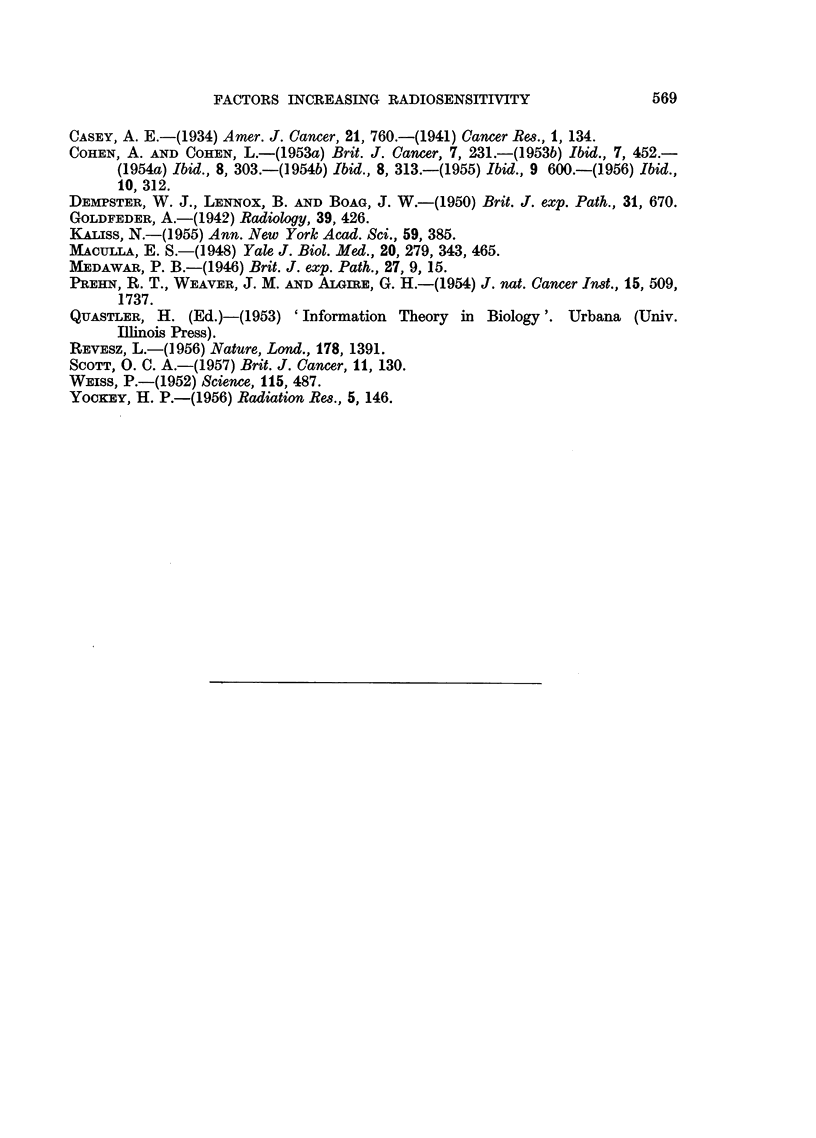# Some Biological Factors Increasing the Radiosensitivity of an Isogenic Mammary Tumour in C3H Mice

**DOI:** 10.1038/bjc.1957.69

**Published:** 1957-12

**Authors:** A. Cohen, L. Cohen


### SOME BIOLOGICAL FACTORS INCREASING THE RADIOSENSITIVITY OF AN ISOGENIC MAMMARY TUMOUR IN C3H MICE

A. COHEN AND L. COHEN

*From the Experimental Oncology Laboratory, Radiation Therapy Department, Johannesburg General Hospital*

Received for publication October 10, 1957

IN a previous paper (Cohen and Cohen, 1956), it was shown that a significant radiosensitization of established C3H mammary tumour isografts could be induced by means of a second inoculation of the hosts with radiation-attenuated tumour fragments prior to treatment. It was noted at the time that the best results were obtained with the largest attenuation dose used (5000 r) provided the attenuated inoculum was given *after* implantation of the unmodified tumour graft, and the treatment of the established growth followed at least two weeks after inoculation of the attenuated material. Some questions concerning the optimal attenuation procedure, the possibility of increasing the effect by " booster " inocula, and the specificity of the sensitizing tissue, were not fully investigated. The following series of experiments were consequently designed to elucidate these points.

### MATERIALS AND METHODS

### *Experimental design*

Young adult male mice of the C3H/Cg strain were used as tumour recipients in all experiments. In order to maintain as uniform a tumour source as possible, a single autogenous mammary adenocarcinoma, arising in a female of the strain, was serially transplanted for ten generations in a group of mice who constituted a pool of tumour donors for all the experimental groups.

Each of 149 recipient experimental mice was inoculated subcutaneously in the right axillary area with a fragment of the tumour introduced with a trocar and cannula. When the isografts became palpable (about 2 or 3 weeks later), the second, or attentuated, inoculum was given subcutaneously into the left flank. The animals were divided, according to the type of second inoculum received, into the 12 experimental categories listed in Table I.

In two groups, attenuated tumour inocula were prepared from isografts minced with a fine scissors and irradiated with 7000 r and 10,000 r respectively, in a plastic welled slide immediately before implantation. At these dose levels there were no " takes " of any of the irradiated tumour implants.

A further two groups were given repeated " booster " inocula of additional tumour fragments attenuated at the two dose levels. In addition to the first attenuated implant given about three weeks before treatment of the tumour *in situ*, the mice were again inoculated one week before, three weeks after treatment, and at monthly intervals thereafter.

In another category, tumour fragments were irradiated with 7000 r and emulsified in an equal volume of Freund's adjuvant complete with *M. butyricum* suspension (" Bacto-Adjuvant ", Difco) prior to inoculation.

Two groups of mice received cytolyzed tumour material. A tumour was finely minced and suspended in twice its volume of distilled water in an aluminium cylinder for 10 minutes at room temperature. It was then frozen at — 70° C. in a solid $CO_2$-alcohol mixture for 5 minutes, and thawed in a water bath at 40° C. for 10 minutes. The freeze-thawing procedure was repeated 5 times. A portion of the cytolyzed material was then irradiated with 7000 r, and fragments inoculated into the left flank of one group of tumour-bearing mice, while an unirradiated portion was similarly inoculated into a second group.

Three other groups of tumour-bearing mice received inocula of skin removed from a newborn C3H mouse, finely minced and unirradiated, or irradiated with 7000 r or 10,000 r respectively, prior to implantation.

Another group was inoculated with spleen removed from an adult C3H mouse, minced into fragments and irradiated with 7000 r prior to implantation.

As controls, a number of tumour-bearing mice from the same series, who had received no additional inoculations of any type, were treated concomitantly with the last of the experimental groups.

Finally, some spontaneous tumours were similarly tested using attenuated autografts. The many difficulties in treating autogenous tumours in this strain were discussed in an earlier paper (Cohen and Cohen, 1953*a*), but it was considered important to investigate whether radiosensitization could be induced also in mice bearing tumours that arose spontaneously from their own tissues. Ten female tumour-bearing mice were accordingly selected; small biopsies of the tumour were removed by surgical excision, and irradiated with 7000 r as described above. The attenuated autografts were then re-inoculated subcutaneously by trocar and cannula. The subsequent procedure was exactly as described above for isografts.

The diameters of the tumours at the time of treatment in the 44 mice receiving isografts attenuated with 10,000 r averaged 13·0 mm., with a standard deviation of 4·7 mm. The 72 tumours in the 7000 r attenuation category had a mean diameter of 12·6 mm., standard deviation 3·5 mm. In the 33 controls, the values were 11·8 and 3·0 mm. respectively. There were no perceptible differences in tumour growth rates among the various groups, and each tumour was irradiated *in situ* with 4200 r when it had attained a diameter of about 1 cm., that is four to seven weeks after implantation, and between two and five weeks after the second inoculation. The expectation of cure at this dose, without preparatory sensitization, is about 0·1 per cent, but significant cure-rates can be obtained when host resistance to the tumour has been stimulated by some experimental manipulation (Cohen and Cohen, 1954*b*). Two or more cures in any single experimental group may then be taken as indicative of a significant induced radiosensitization.

### *Irradiation technique*

As in all previous experiments of this series, rontgen rays generated at 240 kVp, no added filters, HVL 0·34 mm. Cu, were used. For irradiation of the tumour *in situ*, the mice were anaesthetized with intraperitoneal pentobarbital, and using a 2 cm. diameter applicator at 25 cm. FSD, the radiation was directed through the tumour retracted on to a wax block at a dose rate of 500 r/min. The attenuation procedure was carried out by irradiation of minced tissue fragments in a plastic welled slide with the same technical factors, but using a 5 cm. diameter field giving 600 r/min. with full back-scatter, as described in a previous communication (Cohen and Cohen, 1953*a*).

### RESULTS

The results of all the experiments involving isogenic tissue inocula, and their radiosensitizing properties, are shown in Table I. The first two items are from the previously published report, and have been incorporated in the table for comparison with the new data.

TABLE I.—*Radiosensitization of the C3H Mammary Tumour Induced by Radiation-attenuated Isogenic Tissue*

Dose *in situ*, 4200 r

| Type of inoculum | *In vitro* dose (r) | Number of mice | Number cured at 3 months | Number cured at 6 months | 3-month cures (%) |
|---|---|---|---|---|---|
| *Viable tumour isografts | 3,500 | 54 | 7 | 0 | 13 |
| *Arrested tumour isografts | 5,000 | 38 | 19 | 10 | 50 |
| Single tumour implant | 7,000 | 11 | 6 | 4 | 39 |
| Multiple tumour "boosters" | 7,000 | 10 | 4 | 3 | |
| Tumour isograft with adjuvant | 7,000 | 9 | 5 | 3 | |
| †Irradiated tumour cytolysate | 7,000 | 10 | 0 | 0 | |
| Irradiated skin isograft | 7,000 | 22 | 11 | 6 | |
| Irradiated spleen isograft | 7,000 | 10 | 2 | 2 | |
| Single tumour implant | 10,000 | 10 | 1 | 0 | 18 |
| Multiple tumour "boosters" | 10,000 | 22 | 6 | 2 | |
| Irradiated skin isograft | 10,000 | 12 | 1 | 0 | |
| Total irradiated isografts | — | 208 | 62 | 27 | 30 |
| Non-irradiated tumour cytolysate | 0 | 11 | 0 | 0 | 0 |
| Non-irradiated skin isograft | 0 | 11 | 0 | 0 | |
| Uninoculated controls | 0 | 11 | 0 | 0 | |
| §Total controls | — | 33 | 0 | 0 | 0 |

* From previous data (Cohen and Cohen, 1956).

† This material was cytolyzed, that is dead, before irradiation.

§ To this group may be added 97 unattenuated "controls" from previous data, with no "cures" after 4200 r *in situ*.

Comparison shows that the radiosensitizing effect of attenuated isogenic tissue is critically related to the attenuating dose. Whereas only 13 per cent three-month cures were obtained with an attenuation dose of 3500 r, the cure rate approaches a maximum with doses of 5000 and 7000 r, and then falls off sharply at 10,000 r. Of the three groups, totalling 44 mice, that received inocula attenuated at 10,000 r, only 8 (18 per cent) were cured at three months, while in the corresponding three groups, totalling 43 mice, in the 7000 r attenuation category, 21 (50 per cent) were cured at three months. This difference is significant ($p < 0{\cdot}01$) indicating a loss of radiosensitizing ability with increasing radiation dosage. An analogous loss in immunizing ability of excessively irradiated homografts of Sarcoma-180 has been previously described by Goldfeder (1942).

There is reason to believe that with progressive over-dosage, there is not merely a loss of antigenic power in the cells of the inoculum, but the presence of these heavily irradiated cells may actually stimulate the growth of undamaged tumour tissue in the host (Revesz, 1956 ; Scott, 1957) in a manner highly suggestive of the tumour-enhancing properties of lyophilized tissues (Casey, 1934, 1941 ; Kaliss, 1955). The attenuating efficiency relative to dosage is therefore a biphasic curve whose optimum in the region of 6000 r is probably uniquely characteristic of this host-tumour relationship, and, coincidentally or otherwise, happens to be about the same as the median curative dose for the established tumour irradiated *in situ* (Cohen and Cohen, 1953*a*).

Approximately half of the tumours apparently cured at three months eventually recurred. The injection of " booster " inocula, repeated at intervals over the whole period of observation, did not significantly influence either the initial three-month cure rate, nor did it prevent the late recurrences assessed at 6 months.

Freund's adjuvant was used (at the suggestion of J. B. Graham, personal communication, 1957) in an attempt to intensify the sensitizing effect of the attenuated tumour inocula. Under the conditions of this experiment, however, the procedure did not raise the cure rate materially.

It can also be seen that new-born mouse skin isografts irradiated prior to implantation are just as effective as the tumour isografts at the corresponding doses. This result is, presumably, attributable to the common antigens possessed by the two epithelial tissues (Bashford and Murray, 1906 ; Maculla, 1948). A non-epithelial irradiated inoculum such as adult spleen seemed to be somewhat less effective in this regard.

One very obvious trend was the complete loss of radiosensitizing ability when killed (cytolyzed) tumour tissue was inoculated, either unirradiated (0/11 cures) or previously irradiated with 7000 r (0/10 cures).

Non-irradiated skin isografts were ineffective (0/11 cures). There were also no cures in the group of 11 uninoculated controls treated towards the end of this series of experiments, indicating that no inherent increase in radiosensitivity had appeared fortuitously during the serial transmission of this tumour line. The over-all three-month cure rate of 30 per cent among the 208 mice receiving irradiated inocula, compared with no cures among the concomitantly treated controls, furnishes highly significant additional evidence of the radiosensitizing properties ascribed to radiation-attenuated isografts.

In the group of 10 spontaneous mammary tumours in female mice that were inoculated prior to treatment with autografts removed by biopsy and irradiated at 7000 r, there were three cures (not shown in the table). This cure rate is not significantly less than that obtained with isografts, suggesting that, in the C3H mouse at least, conclusions derived from the relatively artificial experimental conditions with grafted tumours, are still valid for the truly autogenous host-tumour relationship.

### DISCUSSION

All the foregoing results consistently support and reinforce the previously proposed immunological hypothesis (Cohen and Cohen, 1956), that the radiation-attenuated isograft had been rendered antigenic to the host, evoking a generalized systemic immune reaction associated with increased radiosensitivity of the established tumour treated *in situ*. If the hypothesis is correct, then ionizing radiation plays a doubly useful role in this type of investigation : it is able to render autogenous and isogenic tumours and tissues, normally incapable of eliciting any immune response, perceptibly antigenic to their hosts ; and, by measuring induced changes in radiosensitivity, it permits the detection of subliminal resistant states or immunizing factors not ordinarily exhibited in the autogenous or isologous relationship. This method has enabled us to induce and detect what amounts to a " systemic radiosensitizing factor " operating in an isologous host-tumour relationship, analogous to the cellular " mobile resistance factor " described by Prehn, Weaver and Algire (1954) in the case of non-isogenic homologous tumour grafts.

The following facts, however, suggest that the phenomenon of induced radiosensitization cannot entirely be explained in conventional immunological terms: Effective " antigen " is apparently confined to physically intact cells attenuated at critical dose levels ; repeated " booster " inocula fail to modify the result, and addition of adjuvants (Freund's) does not enhance the effect ; some other irradiated tissues are just as effective as the attenuated tumour ; and isologous attenuated tumour antigen seems to be effective only when given to a mouse already bearing a tumour.

Since neither isologous nor autogenous transplants can be considered antigenic in the classical sense, it seems that the attenuated inoculum must contain some new material, or antigen modified by the irradiation procedure, capable of influencing a pre-existing balance between the growing tumour and the resistance factors of the host.

One is led to describe this type of interaction in terms of biological " information theory " (Quastler, 1953), which is peculiarly suited to the dynamics of the situation. Self-regulation of protein synthesis and tissue growth (Weiss, 1952) apparently entails a " negative feedback " cycle regulated through the specific tissue " markers " described by Burnet (1954, 1957). Such a cycle is readily " modulated " by extraneous influences. In this case, inoculation with irradiated tissue amounts to injection of disturbing " information " into the system. Ionizing radiation, with its capacity for random disorientation of protein and nucleic acid structure, could well distort the mechanism of normal information-transfer between isogenic tissue and its host, equivalent, in cybernetic terminology, to the superposition of " noise " or " equivocation " (Yockey, 1956) upon the specific " signal ".

When tissues are irradiated *in situ*, the degree of equivocation, that is the noise : signal ratio, becomes sufficient for the cells to lose their specific identity and hence to be regarded as virtually foreign elements subject to the homograft reaction (Medawar, 1946).

All factors inhibiting reticulo-endothelial function in the host, suppressing the homograft reaction, will retard or prevent radiation responses. Total body radiation which delays the onset of homograft immunity (Dempster, Lennox and Boag, 1950) also reduces the radio-curability of tumours (Cohen and Cohen, 1953*b*), and the corticoid hormones (unpublished data) have been shown to possess a similar action. Conversely, activation of host resistance by exhibition of " equivocated " (irradiated) iso-antigens will facilitate radiation reactions and tumour regression.

It should be noted too that the method of cytolysis, whereby the sensitizing ability of the irradiated isograft is destroyed by repeated freeze-thawing, also inactivates the nuclear antigen responsible for eliciting transplantation immunity with homografts (Billingham, Brent and Medawar, 1956). As an interesting corollary, the latent period of about two weeks before the effects of local irradiation become visible, is the same as that for transplantation immunity and many other sensitization reactions.

In previous experiments (Cohen and Cohen, 1954*a*, 1955) with the C3H mammary tumour grown in genetically alien $F_1$ hybrid hosts, and with a mutant tumour line in the homozygous mouse, it was shown that the dose of radiation required to cure a tumour treated *in situ* was determined by immunogenetic differences or antigenic diversification between the tumour and its host. This rule now appears, in view of the foregoing discussion and particularly of the current data on induced radiosensitization, to be a special case of a more general law applicable to all irradiated metazoal tissues and tumours : *Radiosensitivity is a function of antigenic equivocation between the irradiated tissue and its host.*

### SUMMARY

C3H mice bearing isografts of the C3H mammary adenocarcinoma were given one or more inocula of isogenic tumour, skin or spleen, using structurally intact as well as cytolyzed preparations, some unirradiated and others irradiated with varying doses of rontgen rays immediately prior to implantation. The originally established tumours were then irradiated *in situ* with 4200 r, a dose at which the expectation of cure is normally about 0·1 per cent. The cure rate at this dose level in mice inoculated with living minced isogenic tumour tissue or neonatal skin attenuated in the 5000 to 7000 r range, approaches 50 per cent, indicating a very significant radiosensitizing effect. This attenuating dose is similar to the median curative dose for the tumour treated *in situ* in unmodified hosts, and is critical in that the radiosensitizing efficacy falls off sharply with attenuation doses above or below the optimal range.

Repeated "booster" inocula, or the addition of Freund's adjuvant to the attenuated tumour isografts showed no additional effect. Non-irradiated isogenic skin, and dead (cytolyzed) tumour, whether irradiated or not, were completely ineffective.

A group of female mice bearing autogenous mammary tumours and pretreated with attenuated autografts also showed a substantial cure rate at the test dose.

It seems that irradiation of isogenic or autogenous inocula produces antigens capable of sensitizing established tumours to subsequent irradiation. Consideration of the whole series of experiments leads to the conclusion that radiation acts through equivocation of specific identity markers, evoking a virtual homograft reaction against the irradiated isologous or autogenous tissues.

All the necessary facilities for the maintenance of the animals used in these experiments were supplied at the South African Institute for Medical Research, through the generous co-operation of Professor J. F. Murray. The work was supported in part through the Farquhar Bequest Cancer Research Fund. We wish to thank Mrs. Shelley Jacobson for her invaluable technical assistance.

### REFERENCES

Bashford, E. J. and Murray, J. A.—(1906) *Brit. med. J.*, ii, 209.
Billingham, R. E., Brent, L. and Medawar, P. B.—(1956) *Nature, Lond.*, **178**, 514.
Burnet, F. M.—(1954) *Brit. med. J.*, ii, 189.—(1957) *Ibid.*, i, 779, 841.

CASEY, A. E.—(1934) *Amer. J. Cancer*, **21**, 760.—(1941) *Cancer Res.*, **1**, 134.
COHEN, A. AND COHEN, L.—(1953*a*) *Brit. J. Cancer*, **7**, 231.—(1953*b*) *Ibid.*, **7**, 452.—(1954*a*) *Ibid.*, **8**, 303.—(1954*b*) *Ibid.*, **8**, 313.—(1955) *Ibid.*, **9** 600.—(1956) *Ibid.*, **10**, 312.
DEMPSTER, W. J., LENNOX, B. AND BOAG, J. W.—(1950) *Brit. J. exp. Path.*, **31**, 670.
GOLDFEDER, A.—(1942) *Radiology*, **39**, 426.
KALISS, N.—(1955) *Ann. New York Acad. Sci.*, **59**, 385.
MACULLA, E. S.—(1948) *Yale J. Biol. Med.*, **20**, 279, 343, 465.
MEDAWAR, P. B.—(1946) *Brit. J. exp. Path.*, **27**, 9, 15.
PREHN, R. T., WEAVER, J. M. AND ALGIRE, G. H.—(1954) *J. nat. Cancer Inst.*, **15**, 509, 1737.
QUASTLER, H. (Ed.)—(1953) ‘Information Theory in Biology’. Urbana (Univ. Illinois Press).
REVESZ, L.—(1956) *Nature, Lond.*, **178**, 1391.
SCOTT, O. C. A.—(1957) *Brit. J. Cancer*, **11**, 130.
WEISS, P.—(1952) *Science*, **115**, 487.
YOCKEY, H. P.—(1956) *Radiation Res.*, **5**, 146.